# Categorized Affective Pictures Database (CAP-D)

**DOI:** 10.5334/joc.47

**Published:** 2018-09-26

**Authors:** Natali Moyal, Avishai Henik, Gideon E. Anholt

**Affiliations:** 1 Department of Psychology and the Zlotowski Center for Neuroscience, Ben-Gurion University of the Negev, Beer-Sheva, IL

**Keywords:** Emotion and cognition, Stimulus development, Categorisation

## Abstract

Emotional picture databases are commonly used in emotion research. The databases were first based on ratings of emotional dimensions, and the interest in studying discrete emotions led to the categorization of subsets from these databases to emotional categories. However, to-date, studies that categorized affective pictures used confidence intervals in their analysis, a method that provides important data but also results in a high percentage of blended or undifferentiated categorization of images. The current study used 526 affective pictures from four databases and categorized the pictures to discrete emotions in two steps (Pre-testing phase & Experiment 1). First, clinical psychologists were asked to generate emotional labels for each picture, according to the emotion the picture evoked in them. This resulted in the creation of 10 emotional categories. These labels were presented to students who were asked to choose the emotional category that matched the emotion a presented picture evoked in them. Agreement levels on the emotional categories were calculated for each picture, and pictures were categorized according to the most dominant emotion they evoked. The analysis of agreement levels rather than confidence intervals enabled us to provide both dominance of emotional category and agreement in the population regarding the dominance. In Experiment 2, we asked participants to provide ratings of emotional intensity and arousal, in order to provide more detailed information regarding the database. This is the first study to provide agreement levels on the categorization of affective pictures, and may be useful in various studies which aim at generating specific emotions.

Emotional experience has an important, evolutionary role in our lives, since it helps us evaluate the environment and guides our reaction to different situations (Frijda & Mesquita, 1994). The importance of emotional experience makes it a highly significant subject for research. There are two main theories about the evaluation of emotions: the dimensional theory (Fontaine, Scherer, Roesch, & Ellsworth, 2007; Gillioz, Fontaine, Soriano, & Scherer, 2016; Yik, Russell, & Barrett, 1999) and the discrete emotions theory (Darwin, 1872; Ekman & Keltner, 1970; Izard, 1994, 2007). The dimensional theory suggests that emotional experience is multidimensional. However, the question regarding specific dimensions is still under investigation. Most researchers agree that valence (pleasantness) and arousal are basic dimensions, and there is a debate regarding the existence of more dimensions (e.g., dominance, approach-avoidance, etc.; Fontaine et al., 2007; Gillioz et al., 2016; Yik et al., 1999). It was found that emotional dimensions are similar between cultures (Fontaine et al., 2007). The discrete emotions theory suggests that we have a set of distinct emotions that we can recognize and label, and these emotions are cross-cultural (Darwin, 1872; Ekman & Keltner, 1970; Izard, 1994, 2007). There is a complete emotional experience that arises with discrete emotions, which includes bodily sensations, emotional expressions (or the ability to express the emotion), response strategy, and it is evident from infancy (Izard, 2007). These two theoretical frameworks (dimensional and discrete emotions) were investigated separately over the years, but there is now evidence that a combination of these theories can better explain emotional experience (Christie & Friedman, 2004).

The research on emotions uses various types of stimuli (e.g., pictures, words, sounds). To this point, there are several databases for emotional stimuli (e.g., International Affective Pictures System – IAPS (Lang, Bradley, & Cuthbert, 1999); International Affective Digitized Sounds – IADS (Bradley & Lang, 2007); Nencki Affective Pictures System – NAPS (Marchewka, Żurawski, Jednoróg, & Grabowska, 2014); the Geneva Affective Picture Database – GAPED (Dan-Glauser & Scherer, 2011), norms for lemmas (Warriner, Kuperman, & Brysbaert, 2013); Affective Norms for English Words – ANEW (Bradley & Lang, 1999)). The primary classification in emotional databases (IAPS, NAPS, GAPED) is based on the dimensional approach. Classification of the emotional stimuli is according to valence and arousal (for all three databases), dominance (IAPS), approach-avoidance (NAPS), and internal-external norms (GAPED). Importantly, these primary dimensional classifications lack information regarding discrete emotions. Because the effect of specific emotions on behavior and experience have become the focus of various studies (e.g., see Davey, MacDonald, & Brierley, 2008; Duclos et al., 1989; Rusting & Nolen-Hoeksema, 1998; Storbeck & Clore, 2005), information regarding discrete emotions is clearly important. The growing interest in studying discrete emotions and the combination between emotional dimensions and discrete emotions led to the addition of emotional categories to the existing emotional picture databases (e.g., IAPS, NAPS). In the NAPS, for example, Riegel et al. (2016) asked people to rate the intensity of six emotions, which are known as “basic emotions” (Ekman & Keltner, 1970) – happiness, anger, fear, disgust, sadness, and surprise. They found that out of 510 pictures, 72% had a distinct emotional category, 8% were blended and 19% were undifferentiated. Mikels et al. (2005) categorized the IAPS based on labels that were generated in a pilot study according to the emotions the pictures evoked. They found that out of 203 negative pictures, 42% had a distinct emotional category (disgust, fear or sadness), 24% were blended and 34% were undifferentiated. In addition, out of 187 positive pictures, 22% had a distinct emotional category (amusement, awe, contentment, or excitement), 38% were blended and 40% were undifferentiated. Both studies described above used the means of the emotional categories of each picture to determine whether the picture presented a discrete emotion or a blend of various emotions. This method provided a large number of stimuli that were categorized as “undifferentiated”, meaning that they had no dominant emotional category. To our knowledge, there are no studies that classified emotional pictures by discrete emotions based on agreement levels on the emotional category. Agreement levels are an important parameter because they could indicate the probability to which a certain emotion is elicited in response to a specific stimulus.

The aim of the current study was to develop a picture database that contains categorization of affective pictures to discrete emotions, levels of agreement on each emotional category, ratings of emotional intensity, and ratings of arousal. This database can be used in various studies that aim to generate specific emotions. In order to categorize the emotional pictures to discrete emotions, we used both emotion generation (i.e., generating an emotional label to the feeling that the picture evokes) and emotion categorization (i.e., choosing the emotional label that best matches the feeling that the picture evokes, from a list of emotional labels). This two-phase methodology (generation followed by categorization) was used in order to make sure we referred to all relevant emotions that the pictures evoked.

## Pre-testing of Emotional Categories

In the pre-testing of emotional categories, clinical psychologists at various stages of training looked at emotional pictures and generated emotional labels for each picture. This was done in order to ensure that the emotional labels used in the categorization task (Exp. 1) would reflect the emotions the pictures triggered and were not arbitrary. Clinical psychologists at different levels of clinical practice were selected because there is evidence for higher emotional intelligence in therapists compared with patients and prisoners (Schutte et al., 1998). To our knowledge, no comparison between psychologists and the general population was made, but it is reasonable to assume that psychologists have the same, if not better, emotional intelligence than the general population.

### Method

**Materials.** Five-hundred and thirteen pictures were selected for the current experiment. The pictures were chosen from various affective picture sets for psychological research – IAPS (Lang et al., 1999), NAPS (Marchewka et al., 2014), GAPED (Dan-Glauser & Scherer, 2011), and the Berkeley Segmentation Dataset and Benchmark (BSDS300; Martin, Fowlkes, Tal, & Malik, 2007). The BSDS300 dataset is based on segmentation features and does not include emotional information.

**Participants.** Fifteen clinical psychologists (8 females) in various levels of clinical practice (1–13 years of clinical experience, mean = 2.46, *SD* = 2.99) participated in the study (mean age = 30.33 years old, *SD* = 4.35). All participants had normal or corrected-to-normal vision, and no reported history of attention deficit disorder (ADD) or learning disabilities. Participants received 90 NIS (approximately 25$) for participation. All the experiments in this study were approved by the Behavioral Ethics Committee of Ben-Gurion University of the Negev.

**Apparatus.** The study was run on HP ENVY m6 Notebook PC with a 15.6-inch color screen. Visual basic and C# were used for programming, presentation of the stimuli and timing operations. Responses were collected through the computer keyboard.

**Procedure.** After signing a consent form, participants were seated in a quiet room in front of a computer and instructions were presented on the screen, in addition to vocal instructions that were read by the experimenter from a protocol. Participants were informed that unpleasant pictures might appear, and in the case where they felt distressed, they could stop participation in the study at any time. Participants were instructed to look at pictures that appeared in the middle of the screen, and to concentrate on the emotions the pictures evoked in them. Following the picture presentation, a screen with a response box appeared, and participants were asked to write the emotion or emotions they felt while they looked at the picture. Participants were specifically asked to pay attention to what **they** felt, and not to what they thought they should feel or what the characters in the pictures might have felt. Each trial began with the presentation of a fixation cross for 100 ms, followed by a picture that was presented for 10 seconds. Subsequently, a screen with the instruction, “Please write what emotion is evoked in you while looking at the picture”, a response box and a small version of the previously presented picture appeared for 2 minutes or until the participant’s response. Following the response screen, there was an inter-trial interval (ITI) for 2,000 ms. Participants completed six sessions of the experiment with maximum of one-week interval between each two sessions. Each session included 85–86 pictures, in random order for each participant in each session.

### Results

Responses for each participant for each picture were collected. On average, each participant used 1.27 (*SD* = 0.25, range = 0–5) words for each picture. Data regarding the means for each picture and for each participant is provided in the supplementary material, available at: https://osf.io/b4dms/?view_only=f984c0e2ecd04039ac8cbb40ef61b461.

**Figure 1:**
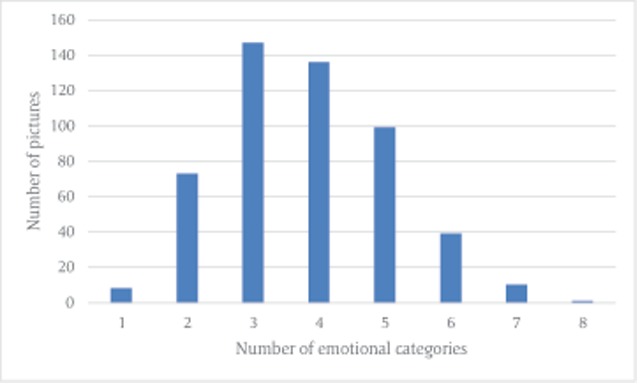
Distribution of frequencies of the number of emotional categories provided to describe pictures.

In order to create the emotional categories, the first author grouped the emotional labels to categories using dictionary definitions (e.g., worry, fear and anxiety were grouped to the category of fear). Each category contained various words with similar dictionary definitions, but the emotional intensity could vary between the words (e.g., fear and terror were grouped to the category of fear). Sixteen emotional categories were used by the psychologists – fear, sadness, disgust, anger, compassion, happiness, love, surprise, pride, peacefulness, hate, shame, regret, hope, jealousy and guilt. After grouping the emotional categories, we calculated the frequency of each emotional category in the responses, and also calculated the most frequent category per image (frequency per image was calculated only when 25% of the psychologists provided the same emotional category). For some pictures, only one emotional category was provided, while for other pictures there were several emotional categories. Figure 1 presents the distribution of frequencies of each number of emotional categories that were provided to describe pictures (e.g., 147 pictures were described by 3 emotional categories).

Based on the emotional labels provided by the clinical psychologists in the pre-test, we used 10 emotional categories in Experiment 1 – fear, sadness, disgust, anger, compassion, happiness, love, surprise, pride and peacefulness. These categories were chosen based on the frequency of appearance in the psychologists’ answers and the most frequent emotional categories for each image (see Table 1). Pride was less frequent than hope in general, but the frequency per image was higher (i.e., more psychologists used pride to describe their emotions in specific pictures than they used hope), meaning there were few pictures in which the most frequent emotional category was pride, and no pictures in which the most frequent category was hope. Hence, we used pride as an emotional category in Experiment 1 and did not use hope. Tab 1 in the supplementary material provides the data regarding each picture in the database – number of words (how many different words were used for each picture), number of categories (how many different categories, including non-emotional categories, were provided for each picture), number of emotional categories, the emotional categories, the main (most frequent) emotional categories for each picture, agreement levels on the main category, and average number of words given to each picture.

**Table 1:** Frequency of the emotional categories.

Emotional category	Image	General	Number of words

Anger	26	148	2
Compassion	62	217	2
Disgust	73	209	3
Fear	76	301	6
Guilt	0	0	1
Happiness	107	204	4
Hate	0	16	3
Hope	0	70	3
Jealousy	0	41	1
Love	16	152	3
Peacefulness	24	111	3
Pride	2	47	1
Regret	0	4	1
Sadness	63	234	7
Shame	0	23	2
Surprise	4	160	5

*Note*. Image – the number of pictures in which the specific category was the most frequent. General – the number of times the category was provided by the psychologists. Number of words – How many words were used to describe the emotional category.

## Experiment 1

The aim of the current experiment was to examine to which emotional category each picture belonged, and what the agreement levels for this category were (i.e., whether there was a consensus). In the current experiment, students conducted an emotion categorization task based on the emotional categories we found in the pre-testing phase.

## Experiment 1a

### Method

**Materials.** The materials were identical to those in the pre-testing stage.

**Participants.** One-hundred and eight students from Ben-Gurion University of the Negev participated in the study. Eight students did not complete the task. Hence, 100 students (56 females, mean age = 24.47 years old, *SD* = 2.35) participated and completed the study. All participants had normal or corrected-to-normal vision, and no reported history of ADD or learning disabilities. Participants received 60 NIS (approximately 16$) for participation in the study.

**Apparatus.** The experiment was run on an IBM-PC with a 22-inch color screen monitor. Open-sesame (Mathôt, Schreij, & Theeuwes, 2012) was used for programming, presentation of the stimuli and timing operations. Responses were collected using the computer mouse.

**Figure 2:**
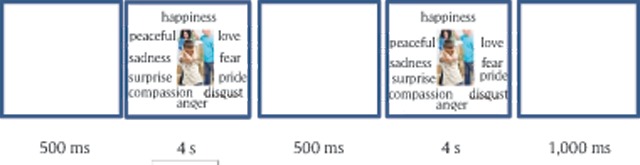
An example trial from Experiment 1.

**Procedure.** Participants were seated in groups of 2–12 students in a computer room, and signed consent forms. Verbal instructions were given prior to the beginning of the experiment. Participants were asked to look at the pictures that were presented on the screen, pay attention to the emotion that each picture evoked in them, and choose the emotional category that matched the emotion they felt. Each picture was presented twice, and participants were asked to choose at the first presentation, the most dominant, salient, or first emotion they felt. At the second presentation of the picture, participants were asked to choose another emotion that was evoked in them, if there was another emotion. If they only noticed one emotion, participants could skip the second categorization. Each trial began with a blank screen that was presented for 500 ms, followed by a screen with a picture and 10 emotional categories around it, which was presented for 4,000 ms. Then, a blank screen appeared for 500 ms, followed by the same picture and categorization screen, which was again presented for 4,000 ms. The trial ended with ITI of 1,000 ms (an example of a trial is presented in Figure 2). The order of the categories was fixed, meaning that the categories were always presented at the same place on the screen, and in each categorization screen the starting point of the mouse was in the middle of the screen, an equal distance from all the emotional labels. Participants completed 2 sessions of the task (with 256 picturesin one session and 257 pictures in the other session, in random order), with a one-week interval between the sessions. The order of the sessions was counter-balanced between participants.

### Results

**Figure 3:**
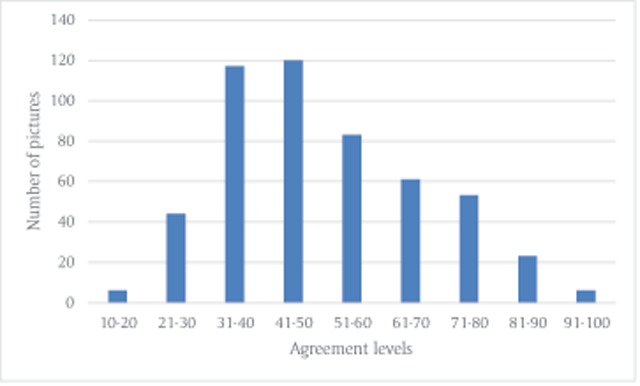
Distribution of number of pictures according to agreement levels in Experiment 1a.

**First categorization.** Pictures were classified according to the most frequent emotional category given by the participants, and agreement levels on this category were calculated for each picture (by dividing the number of participants who chose this category by the total number of participants). In order to examine the reliability of the pictures, we examined the relationship between the agreement levels in the current experiment and the data from the pre-testing phase. As expected, we found negative correlations between agreement levels and number of words (*r* = –0.43, *p* < 0.01), number of categories (*r* = –0.34, *p* < 0.001), and number of emotional categories (*r* = –0.38, *p* < 0.01). We also found a positive correlation between agreement levels in the current experiment and agreement levels in the pre-test (*r* = 0.44, *p* < 0.01) (see Table 2). In addition to the main categorization, pictures with agreement of 21% or more on another category were classified as belonging to the most frequent category but data on this additional category was also available (e.g., a picture with 40% agreement on sadness and 25% agreement on fear was categorized as evoking sadness and evoking fear was an additional category). Since we had five positive categories and five negative categories, 21% was the number indicating that categorization was not random. For data regarding the number of pictures in each category, and amount of pictures with an additional category in each main category, see Table 3. In order to further investigate the degree of convergence between participants, we used the H statistic. This statistic examines name agreement and is usually used in studies that aim to examine convergence in response to names of objects (Dimitropoulou, Duñabeitia, Blitsas, & Carreiras, 2009; Duñabeitia et al., 2018; Snodgrass & Vanderwart, 1980). An H statistic of 0 means 100% agreement regarding the name (or the emotional category in the current study). Higher values of the H statistic indicate lower agreement on the emotional category. We used the same formula as in the study of Snodgrass and Vanderwart (1980). The range of the H statistic in the current experiment was 0.23 – 2.65 (Mean = 1.63, *SD* = 0.46; the data regarding the H statistic of each picture is available in Tab 3 in the supplementary material). The distribution of the number of pictures for the various agreement levels is presented in Figure 3. Data regarding the agreement levels of each picture for the entire sample, and data for males and females is available in the supplementary material (Tabs 3–5).

**Table 2:** Correlations between parameters from the pre-testing phase and agreement levels from Experiment 1.

	Number of words	Number of categories	Number of emotional categories	Agreement levels psychologists

Agreement levels Exp. 1a	–0.43**	–0.34***	–0.38**	0.44**
Agreement levels Exp. 1b	–0.44**	–0.35***	–0.39**	0.45**

*Note*. ** *p* < 0.01, *** *p* < 0.001.

**Table 3:** Distribution of pictures to emotional categories in Exp. 1a.

Emotional category	Number of pictures (% of 513 pictures)	Number of pictures with an additional category (% of pictures in the category)

Anger	20 (0.03)	14 (0.7)
Compassion	20 (0.03)	19 (0.95)
Disgust	89 (0.17)	32 (0.37)
Fear	90 (0.17)	56 (0.62)
Happiness	112 (0.21)	47 (0.41)
Love	22 (0.04)	16 (0.72)
Peacefulness	55 (0.1)	28 (0.5)
Pride	17 (0.03)	9 (0.52)
Sadness	86 (0.16)	61 (0.7)
Surprise	2 (0.003)	0 (0)

**Second categorization.** The second categorization was calculated the same way as the primary categorization was, that is, according to agreement levels. However, in the second categorization, participants were instructed to choose an emotional category only if they felt an additional emotion while looking at the pictures. Hence, the agreement levels were lower compared with the first categorization, since not all the participants responded to the second categorization. Data regarding categorization and agreement levels is available in Tabs 3–5 in the supplementary material.

## Experiment 1b

Experiment 1a provided a small number of pictures triggering anger. Because many researchers use negative pictures in their research, and we wanted the CAP-D to provide enough pictures, we ran another experiment designed to find more pictures for the anger category.

### Method

**Materials.** All 513 pictures from the pre-testing phase and Experiment 1a were used. Based on the characteristics of the pictures that were categorized as anger in Experiment 1a, we found 13 additional pictures from the IAPS (Lang et al., 1999) with the same characteristics. Hence, 526 pictures were used in the current study.

**Participants.** Thirty-eight students from Ben-Gurion University of the Negev participated in the study. Four students did not complete the task and one was excluded from the analysis due to a high percentage of missing values (49.23% missing values in the first categorization). Hence, 33 participants (17 females) completed the study (mean age = 24.15 years old, *SD* = 1.7). All participants had normal or corrected-to-normal vision, and no reported history of ADD or learning disabilities. Participants received 60 NIS (approximately 16$) for participation in the study.

**Apparatus.** The apparatus was the same as in Experiment 1a.

**Procedure.** The procedure was similar to Experiment 1a except for the number of pictures. In Experiment 1b, participants were presented with 526 pictures (263 in each session, in random order), instead of 513 pictures.

### Results

**Figure 4:**
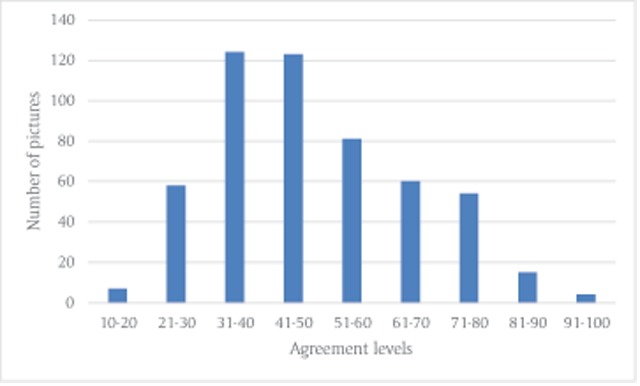
Distribution of number of pictures according to agreement levels in Experiment 1b.

First, we wanted to verify that the current sample of participants was similar to the sample of Experiment 1a. We calculated agreement levels as described in Experiment 1a and used Pearson correlations to assess the similarity between the agreement levels in both samples. The correlation between agreement levels (each picture had two agreement levels) when all pictures were included (regardless of the emotional category) was *r* = 0.86 (*p* < .001). Four-hundred and nineteen pictures were classified as having the same emotional category in both samples. The correlation between agreement levels in these pictures was *r* = 0.85 (*p* < .001). Since the correlations were sufficiently high, we combined the two samples into one sample and the final classification of pictures by emotional categories was based on a sample of 133 participants. For data regarding the number of pictures in each category, and amount of pictures with an additional category in each main category, see Table 4. The H statistic range for 513 pictures, which were categorized by 133 subjects, is 0.29–2.61 (Mean = 1.63, *SD* = 0.44). From the additional 13 pictures, 4 were classified as fear (1 of them had an additional category), 3 were classified as disgust, 5 were classified as anger and 1 was classified as sadness. The H statistic range for the additional 13 pictures, which were categorized by 33 subjects, was 0.92–2.17 (Mean = 1.74, *SD* = 0.32). Data regarding the agreement levels and the H statistic of the entire sample, and data for males and females is available in the supplementary material (Tabs 6–9). In addition, data regarding the correlations with the pre-test parameters is provided in Table 2. The distribution of number of pictures for the various agreement levels for 133 subjects is presented in Figure 4.

**Table 4:** Distribution of pictures to emotional categories in Exp. 1b.

Emotional category	Number of pictures (% of 513 pictures)	Number of pictures with an additional category (% of pictures in the category)

Anger	25 (0.04)	14 (56)
Compassion	13 (0.02)	11 (0.84)
Disgust	88 (0.16)	29 (0.32)
Fear	95 (0.18)	61 (0.64)
Happiness	114 (0.21)	42 (0.36)
Love	22 (0.04)	16 (0.72)
Peacefulness	56 (0.1)	26 (0.46)
Pride	15 (0.02)	6 (0.4)
Sadness	97 (0.18)	62 (0.63)
Surprise	1 (0.001)	0 (0)

## Experiment 2

Our previous studies provided information on discrete emotions for each picture in the database. The aim of the current study was to provide information regarding emotional dimensions, so that our database would include wide-scale information for each picture, which in turn, would help researchers choose emotional stimuli based on relevant parameters for them. In this study, we used scales of emotional intensity and arousal. An emotional intensity scale rather than valence was used for a few reasons. First, when creating the emotional labels in the pre-testing, we noticed that participants’ responses seemed to be contingent on the intensity of the emotions described (e.g., in some pictures participants reported they felt fear and in others they reported feeling terror, or anger vs. fury). Second, for most pictures, we already have two categorizations for negative vs. positive (in the current study we have emotional labels and in the IAPS and NAPS, which are 79% of the pictures, there are valence ratings). Third, the emotional intensity scale was found to be more informative, since it is wider (scale of 1 to 9, and not 1–4 for negative and 6–9 for positive in the valence scale) and enables participants to be more precise in describing the intensity of their feelings. This, in turn, will enable researchers to choose pictures that evoke a specific emotion and specific intensity.

### Method

**Materials.** The materials were the same as in Experiment 1b.

**Participants.** Fifty-eight students from Ben-Gurion University of the Negev participated in the study. All students were recruited personally via e-mail after completing Experiment 1a. One participant did not complete the task and was removed from the analysis. Hence, 57 participants (30 females) participated and completed the task (mean age = 24.75 years old, *SD* = 2.17). Participants received 90 NIS (approximately 25$) for participation in the study.

**Apparatus.** The experiment was run on an IBM-PC with a 22-inch color screen monitor. E-prime software was used for programming, presentation of the stimuli and timing operations. Responses were collected using the computer keyboard.

**Procedure.** Participants were seated alone or in groups of 2–12 students in a computer room, and signed consent forms. Verbal instructions were given prior to the beginning of the experiment. Participants were asked to look at the pictures that were presented on the screen and rate each picture on two scales – intensity and arousal. The instructions for the experiment were: *Hello and thank you for participating in the study. During the current experiment you will be presented with pictures, and you will be asked to rate each picture on two scales. The first scale is the intensity scale. Please indicate how intense was the emotion you felt when you saw the picture. On this scale, 1 represents pictures that do not evoke emotions in any intensity, or a neutral feeling, and 9 represents pictures that evoke an emotion that is highly intense. The emotion that arises can be positive or negative. We ask you to pay attention to the intensity of the emotion that arises in you and respond as fast and as accurately as possible. The second scale is the arousal scale. Please indicate how arousing was the picture – to what extent it made you feel excited, stimulated, wide-awake or physically aroused. On this scale, 1 represents pictures that made you feel relaxed, calm, sleepy, unaroused, and 9 represents pictures that made you feel highly aroused. In both scales, please choose the number that best represents your feeling. There are no correct answers; we want to know how these pictures make people feel, so when we say ‘accurately’ we are referring to your feeling*. The instructions for the arousal scale were based on the instructions that were given in the IAPS (Lang et al., 1999). Each trial began with a picture that was presented for 4,000 ms, followed by an intensity scale that was presented for 5,000 ms. Then, an arousal scale was presented for 5,000 ms. Participants completed 2 sessions of the task (with 263 pictures in each session), with a one-week interval between the sessions. The order of the sessions was counter-balanced between participants. Due to a problem with the E-prime software, the order of the pictures was fixed in each session. When the problem was found, we decided to reverse the order of the pictures for all the subsequent participants. Hence, 12 participants completed the experiment in reversed order of the pictures, compared with the other 45 participants.

### Results

In order to verify that the results were not affected by the order in which the pictures were presented, the correlation between the ratings in both versions of presentation was examined. The correlation between the intensity ratings was 0.82 (*p* < .001), and the correlation between the arousal ratings was 0.85 (*p* < .001). Hence, we merged the results from both versions. Next, we calculated means and standard deviations of intensity and arousal ratings for each picture, for all the participants, as well as for males and females (for the detailed statistics please see Tab 9 in the supplementary material). The correlation between intensity and arousal ratings was examined and it was significant (*r* = 0.93, *p* < .001).

## General Discussion

The aim of the current study was to create a database of emotional pictures that would include parameters of both discrete emotions and affective dimensions. This database will enable researchers to study specific emotions, compare effects of different emotions, and use not only valence but also emotion intensity.

Although there are studies that allocated emotional pictures (e.g., IAPS, NAPS) to discrete emotions (Mikels et al., 2005; Riegel et al., 2016), this is the first study that provides agreement levels on the categorization of stimuli to discrete emotions. Agreement levels can help researchers choose stimuli that evoke specific emotions in high vs. low percentages of the population, according to the nature of the study. Previous research (Mikels et al., 2005; Riegel et al., 2016) has reported large numbers of “blended” or “undifferentiated” pictures. We think that this is due to the methodology used in those studies. Those studies asked participants to grade how strong a given picture indicated a given emotion. These grades were averaged and a given picture was assigned to a specific emotion when its grade for that emotion did not overlap with its grade for other emotions. Because emotion grades for many pictures overlapped, they were categorized as undifferentiated or blended. In contrast, we asked participants to categorize pictures according to the dominant emotion and computed agreement levels for each picture. As can be seen in Tab 9 in the supplementary material, quite a few pictures have a high agreement level and can be thought of as categorized to a specific emotion. Other pictures may have lower agreement levels and may be thought of as blended as they have a sizeable agreement level on more than one emotion. In addition, due to the analysis of means and confidence intervals, both the IAPS and NAPS have relatively small samples of pictures for each emotion, and the positive emotions in the two databases are completely different. The small samples of positive pictures and the inconsistency in the emotional labels might limit researchers who intend to study positive emotions. Our database expands the knowledge that exists in the literature regarding these two highly useful picture systems and provides comparable information regarding emotional categories on pictures from the IAPS and NAPS, in addition to other picture systems (GAPED, BSDS300).

The current study has several limitations. First, some of the emotional categories (e.g., surprise, anger, compassion) have small numbers of pictures. This underrepresentation of certain categories can be explained in several ways. First, it might be that we did not choose enough pictures to represent each category. It is also possible that the emotional picture systems that are available for psychological studies have underrepresentation of certain emotions, since the pictures were not selected based on emotional categories. Another explanation that is in line with previous results and was suggested in other categorization papers (Mikels et al., 2005, Riegel et al., 2016) is that surprise and anger are less likely to arise in response to pictures. When we examined the data, we found that surprise and anger were more common as an additional category. In the pre-testing phase, surprise was evoked 160 times, but was the main category only 4 times, and anger was evoked 148 times but was the main category 26 times. In Exp. 1b, anger was reported as a secondary emotion 63 times (as opposed to 25 times as a main category). Surprise was reported less than other categories even as a secondary emotion, and this may suggest that it is less meaningful in the emotional experience when one is viewing emotional scenes and needs to choose only two emotional labels to describe one’s experience.

Another limitation of the current study is the high correlation between the dimensions of intensity and arousal. We chose to use intensity in order to expand the ability of the participants to be precise regarding their subjective feeling regarding each picture by using a wider scale (9 options instead of 3–4 in the valence scales). To the best of our knowledge, there is not enough existing data regarding factors that determine emotional intensity. Hence, it is hard to conclude from the current study whether the high correlation between intensity and arousal is unusual or if it reflects the relationship between these two scales.

A third limitation is that the values of the H statistic seem to be relatively high compared with the values in other studies that used this statistic (Dimitropoulou et al., 2009; Duñabeitia et al., 2018; Snodgrass & Vanderwart, 1980). However, it is important to note that in previous studies, participants were asked to name known objects (e.g., fruits, animals, vehicles, etc.), while in the current study, participants were asked to choose one out of ten categories to describe their emotions when viewing ambiguous scenes. Hence, it is not surprising that the values of the H statistic show more varied responses than when object names are probed.

To conclude, to the best of our knowledge, the present study is the first to provide information regarding categorization of emotional pictures according to agreement levels in the population. This data is highly important in studies that aim at evoking discrete emotions, or studies that aim at creating variance in the level of certainty regarding the emotion that a certain picture evokes, and uses more ambiguous pictures. In addition, the information regarding intensity and arousal makes it now possible to match pictures on these ratings, in addition to agreement levels on the discrete emotions.

## Data Availability

Data files may be downloaded here: https://osf.io/b4dms/?view_only=f984c0e2ecd04039ac8cbb40ef61b461.

## References

[1] Bradley, M. M., & Lang, P. J. (1999). Affective norms for English words (ANEW): Instruction manual and affective ratings, 30(1), 25–36. Technical Report C-1, Gainesville, FL The Center for Research in Psychophysiology, University of Florida.

[2] Bradley, M. M., & Lang, P. J. (2007). International Affective Digitized Sounds (2nd Edition; IADS-2): Affective ratings of sounds and instruction manual (Technical Report No. B-3). Gainesville, FL: University of Florida, NIMH Center for the Study of Emotion and Attention.

[3] Christie, I. C., & Friedman, B. H. (2004). Autonomic specificity of discrete emotion and dimensions of affective space: A multivariate approach. International Journal of Psychophysiology, 51(2), 143–153. DOI: 10.1016/j.ijpsycho.2003.08.002 14693364

[4] Dan-Glauser, E. S., & Scherer, K. R. (2011). The Geneva affective picture database (GAPED): A new 730-picture database focusing on valence and normative significance. Behavior Research Methods, 43, 468–477. DOI: 10.3758/s13428-011-0064-1 21431997

[5] Darwin, C. (1872). The expression of emotion in animals and man London: Methuen DOI: 10.1037/10001-000

[6] Davey, G. C. L., MacDonald, B. A., & Brierley, L. (2008). The effect of disgust on anxiety ratings to fear-relevant, disgust-relevant and fear-irrelevant stimuli. Journal of Anxiety Disorders, 22, 1347–1354. DOI: 10.1016/j.janxdis.2008.01.015 18343631

[7] Dimitropoulou, M., Duñabeitia, J. A., Blitsas, P., & Carreiras, M. (2009). A standardized set of 260 pictures for Modern Greek: Norms for name agreement, age of acquisition, and visual complexity. Behavior Research Methods, 41(2), 584–589. DOI: 10.3758/BRM.41.2.584 19363201

[8] Duclos, S. E., Laird, J. D., Schneider, E., Sexter, M., Stern, L., & Van Lighten, O. (1989). Emotion-specific effects of facial expressions and postures on emotional experience. Journal of Personality and Social Psychology, 57, 100–108. DOI: 10.1037/0022-3514.57.1.100

[9] Duñabeitia, J. A., Crepaldi, D., Meyer, A. S., New, B., Pliatsikas, C., Smolka, E., & Brysbaert, M. (2018). MultiPic: A standardized set of 750 drawings with norms for six European languages. Quarterly Journal of Experimental Psychology, 71(4), 808–816. DOI: 10.1080/17470218.2017.1310261 28326995

[10] Ekman, P., & Keltner, D. (1970). Universal facial expressions of emotion. California Mental Health Research Digest, 8, 151–158.

[11] Fontaine, J. R. J., Scherer, K. R., Roesch, E. B., & Ellsworth, P. C. (2007). The world of emotions is not two-dimensional. Psychological Science, 18, 1050–1057. DOI: 10.1111/j.1467-9280.2007.02024.x 18031411

[12] Frijda, N. H., & Mesquita, B. (1994). The social roles and functions of emotions In: Kitayama, S., & Markus, H. R. (eds.) Emotion and culture: Empirical studies of mutual influence, 51–87. Washington, DC, US: American Psychological Association DOI: 10.1037/10152-002

[13] Gillioz, C., Fontaine, J. R. J., Soriano, C., & Scherer, K. R. (2016). Mapping emotion terms into affective space. Swiss Journal of Psychology, 75, 141–148. DOI: 10.1024/1421-0185/a000180

[14] Izard, C. E. (1994). Innate and universal facial expressions: Evidence from developmental and cross-cultural research. Psychological Bulletin, 115, 288–299. DOI: 10.1037/0033-2909.115.2.288 8165273

[15] Izard, C. E. (2007). Basic emotions, natural kinds, emotion schemas, and a new paradigm. Perspectives on Psychological Science, 2, 260–280. DOI: 10.1111/j.1745-6916.2007.00044.x 26151969

[16] Lang, P. J., Bradley, M. M., & Cuthbert, B. N. (1999). International Affective Picture System (IAPS): Technical manual and affective ratings. Gainesville: University of Florida, Center for Research in Psychophysiology.

[17] Marchewka, A., Żurawski, Ł., Jednoróg, K., & Grabowska, A. (2014). The Nencki Affective Picture System (NAPS): Introduction to a novel, standardized, wide-range, high-quality, realistic picture database. Behavior Research Methods, 46, 596–610. DOI: 10.3758/s13428-013-0379-1 23996831 PMC4030128

[18] Martin, D., Fowlkes, C., Tal, D., & Malik, J. (2007). The Berkeley Segmentation Dataset and Benchmark University of California, Berkeley http://www.cs.berkeley.edu/projects/vision/grouping/segbench.

[19] Mathôt, S., Schreij, D., & Theeuwes, J. (2012). OpenSesame: An open-source, graphical experiment builder for the social sciences. Behavior Research Methods, 44, 314–324. DOI: 10.3758/s13428-011-0168-7 22083660 PMC3356517

[20] Mikels, J. A., Fredrickson, B. L., Larkin, G. R., Lindberg, C. M., Maglio, S. J., & Reuter-Lorenz, P. A. (2005). Emotional category data on images from the international affective picture system. Behavior Research Methods, 37, 626–630. DOI: 10.3758/BF03192732 16629294 PMC1808555

[21] Riegel, M., Żurawski, Ł., Wierzba, M., Moslehi, A., Klocek, Ł., Horvat, M., Marchewka, A., et al. (2016). Characterization of the Nencki Affective Picture System by discrete emotional categories (NAPS BE). Behavior Research Methods, 48, 600–612. DOI: 10.3758/s13428-015-0620-1 26205422 PMC4891391

[22] Rusting, C. L., & Nolen-Hoeksema, S. (1998). Regulating responses to anger: Effects of rumination and distraction on angry mood. Journal of Personality and Social Psychology, 74, 790–803. DOI: 10.1037/0022-3514.74.3.790 9523420

[23] Schutte, N. S., Malouff, J. M., Hall, L. E., Haggerty, D. J., Cooper, J. T., Golden, C. J., & Dornheim, L. (1998). Development and validation of a measure of emotional intelligence. Personality and individual differences, 25(2), 167–177. DOI: 10.1016/S0191-8869(98)00001-4

[24] Snodgrass, J. G., & Vanderwart, M. (1980). A standardized set of 260 pictures: norms for name agreement, image agreement, familiarity, and visual complexity. Journal of Experimental Psychology: Human Learning and Memory, 6(2), 174–215. DOI: 10.1037/0278-7393.6.2.174 7373248

[25] Storbeck, J., & Clore, G. L. (2005). With sadness comes accuracy; with happiness, false memory: Mood and the false memory effect. Psychological Science, 16, 785–791. DOI: 10.1111/j.1467-9280.2005.01615.x 16181441

[26] Warriner, A. B., Kuperman, V., & Brysbaert, M. (2013). Norms of valence, arousal, and dominance for 13,915 English lemmas. Behavior Research Methods, 45(4), 1191–1207. DOI: 10.3758/s13428-012-0314-x 23404613

[27] Yik, M. S., Russell, J. A., & Barrett, L. F. (1999). Structure of self-reported current affect: Integration and beyond. Journal of Personality and Social Psychology, 77, 600–619. DOI: 10.1037/0022-3514.77.3.600

